# Challenges in the Development of Novel Therapies for Thoracic Malignancies

**DOI:** 10.14789/ejmj.JMJ25-0017-R

**Published:** 2025-08-30

**Authors:** KEN TAJIMA

**Affiliations:** 1 Department of Respiratory Medicine, Juntendo University Graduate School of Medicine, Tokyo, Japan; 1 Department of Respiratory Medicine, Juntendo University Graduate School of Medicine, Tokyo, Japan

**Keywords:** thoracic malignancies, epigenetics, senescence, lung neoplasms

## Abstract

This study explores the potential of targeting epigenetics and cellular senescence as novel therapeutic strategies for cancer, with a focus on thoracic malignancies. Despite significant advances in cancer treatment, including molecularly targeted therapies and immune checkpoint inhibitors, challenges such as drug resistance and side effects remain. Recent research has highlighted the role of epigenetic abnormalities, such as histone modifications, in regulating gene expression and promoting cancer progression. In particular, inhibition of SETD1A, a histone methyltransferase involved in H3K4 methylation, has been shown to suppress tumor growth and metastasis in vivo. In addition, SETD1A inhibition also induces cellular senescence as evidenced by cell cycle arrest and increased expression of senescence markers. These findings suggest that the epigenetic regulation of tumor suppressor genes and the induction of cellular senescence could represent a new approach to cancer therapy. While traditional anticancer drugs primarily induce apoptosis, targeting cellular senescence may provide a more stable, long-term suppression of tumor growth. These findings highlight the need for further research into epigenetic mechanisms and their role in cancer progression. A better understanding of these processes may lead to the development of more effective, targeted therapies for the treatment of thoracic and other malignancies.

## Introduction

The history of cancer drug therapy began in the mid-1900s with nitrogen mustard, a compound derived from mustard gas^1)^. These early cytotoxic agents targeted DNA synthesis and cell proliferation, but also affected normal cells, causing significant side effects. While these drugs dominated cancer treatment for decades, major advances in the 1990s changed the landscape, especially for thoracic malignancies.

A breakthrough occurred when researchers recognized that carcinogenesis is a multi-step process involving oncogenes and tumor suppressor genes^2)^. This led to the development of molecularly targeted therapies, small molecules, that inhibit oncogenes^3)^. Another key discovery was PD-1 and PD-L1, immune checkpoint molecules that enable cancers to evade the immune response^4)^. Immune checkpoint inhibitors, which restore immune cell activity, are now in clinical use and have significantly improved patient outcomes^5, 6)^.

Despite these advances, challenges such as drug resistance and side effects remain. Recently, epigenetic abnormalities have gained attention as contributing to widespread gene dysregulation in cancer^7)^. Epigenetic mechanisms, such as histone modifications, may offer potential new therapeutic targets^8-10)^. Additionally, most anticancer drugs induce apoptosis but do not cure cancer. Cellular senescence, another anti-cancer mechanism, may be a novel therapeutic approach. Epigenetics also plays a role in inducing and maintaining senescence in cancer cells^11)^.

Focusing on epigenetics and thoracic malignancies, my research has investigated how histone methylation regulates tumor suppressor gene expression and cellular senescence. In this paper, I will discuss the potential of novel cancer therapies based on epigenetics and cellular senescence.

## Focus on epigenetics

Epigenetics is often described as the mechanism by which gene expression is regulated without altering the underlying DNA sequence. I would like to begin by explaining why I became particularly interested in this field. While conducting research abroad at the MGH Cancer Center, where I conducted research as part of my study abroad program, I worked in a lab focused on the tumor suppressor BTG2. BTG2 is reported to function as a direct downstream target of p53 and plays a role in cell cycle regulation. My main research at the time mainly focused on analyzing the functional aspects of BTG2. From the outset, I was intrigued by a remarkable observation: while genetic alterations are well-known mechanisms for tumor suppressor inactivation - p53, for example, is widely reported to harbor numerous mutations - there were virtually no reports of mutations in BTG2. This led me to hypothesize that BTG2 is regulated more by epigenetic mechanisms rather than genetic modifications.

## Screening histone methyltransferases using an shRNA library

**Figure 1:**
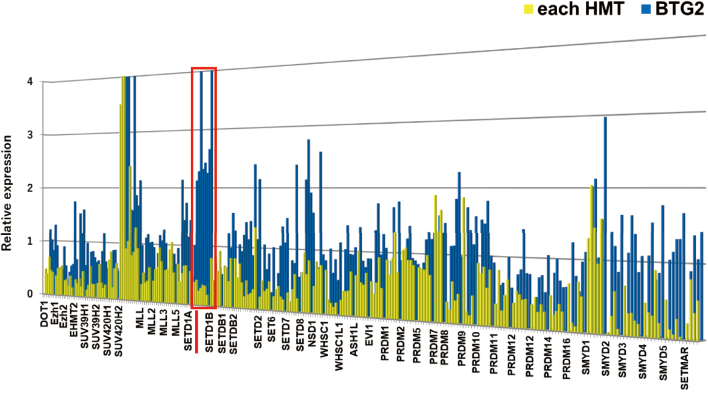
Knockdown of SETD1A Specifically Induces BTG2 Expression A lentiviral shRNA screen targeting H3K4 KMTs in MDA-MB-231 cells demonstrates that depletion of SETD1A specifically induces BTG2 expression compared with control cells infected with pLKO. The fold change in BTG2 and KMT expression after 72 hours of viral infection is presented, with control levels normalized to 1.

To identify regulatory factors of BTG2, I focused on epigenetics, specifically histone modifications, and performed a screen using an shRNA library. After searching the literature for genes potentially involved in histone methylation and targeted 43 histone methyltransferases (HMTs). I then performed knockdown experiments using over 200 shRNAs. As a result of this screening, SETD1A, an H3K4-specific lysine methyltransferase (KMT), emerged as a candidate (Figure 1). SETD1A forms the COMPASS complex with seven other proteins and catalyses the mono-, di- and tri-methylation of H3K4^12)^.

## Antitumor effects by targeting epigenetics

**Figure 2:**
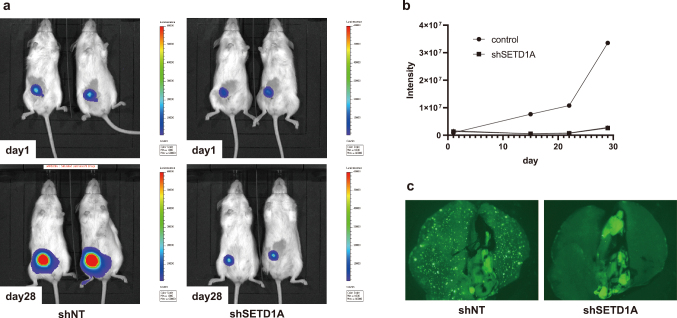
SETD1A regulates tumorigenesis (a) Serial bioluminescence imaging of shNT and shSETD1A-MDA-MB-231 tumor-bearing mice. Luciferase-expressing MDAMB-231 cells infected with shNT or shSETD1A lentiviruses were inoculated into the mammary fat pads of immunocompromised mice. (b) Tumor growth was assessed using bioluminescence imaging. (c) GFP-labeled MDA-MB-231 cells infected with shNT or shSETD1A lentiviruses were inoculated into the mammary fat pads of immunocompromised mice. Liver metastases were identified by tracking GFP signals using a stereomicroscope.

Inhibition of SETD1A resulted in several changes, the most notable of which were observed in in vivo experiments. First, breast cancer cell lines were infected with a GFP-luciferase viral vector. Then, the cells were infected with either a shSETD1A viral vector or a control vector. The cells were then transplanted subcutaneously into mice and tumor size was measured over time. The results showed that tumor growth was significantly suppressed in the shSETD1A group (Figure 2a and 2b). Additionally, upon sacrificing the mice on day 28 and examining their lungs under a stereomicroscope, numerous GFP signals were detected in the control group but not in the shSETD1A group (Figure 2c). These results suggest that inhibition of SETD1A has anti-tumor effects.

## Induction of cellular senescence

**Figure 3:**
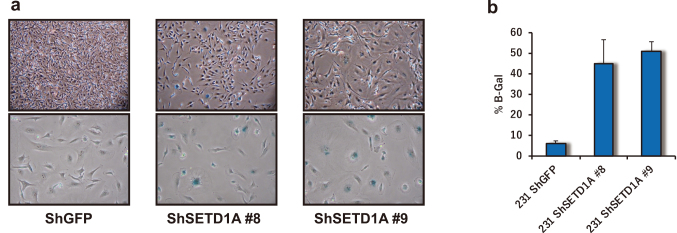
SETD1A depletion induces senescence (a) Images of β-gal-stained shGFP and shSETD1A (#8 and #9) MDA-MB-231 cells are shown. (b) The bar graph illustrates the percentage of β-gal-positive cells in MDA-MB-231 cultures infected with shSETD1A (#8 and #9) or shGFP.

I would like to shift the discussion to the mechanisms underlying the antitumor effects observed with SETD1A inhibition. Suppressing SETD1A resulted in a significant reduction in cell proliferation compared to the control group. To investigate this further, we examined the expression of cell cycle related factors. Following SETD1A suppression, we observed increased expression of p21 and p27, which suggests that the cell cycle was arrested. However, to determine whether apoptosis was induced, we examined the expression of cleaved caspase and PARP. Compared to the control group, in which apoptosis was induced by UV treatment, no increase in the expression of these markers was observed in SETD1A-suppressed cells^11)^. These results suggest that apoptosis was not induced. Given the cell cycle arrest, we performed SA-β-gal staining and found that cells with SETD1A suppression were significantly stained (Figure 3a and 3b). This clearly shows that SETD1A inhibition induces cellular senescence.

## Conclusion

The results of this study suggest the potential of therapies that target epigenetics and cellular senescence. However, it has also been shown that senescent cells, which are expected to be eliminated by immune cells, can persist and exhibit a phenotype known as the senescence-associated secretory phenotype (SASP). These cells secrete cytokines and chemokines such as IL-1β, IL-6, and IL-8, which trigger inflammation and promote tumor growth. Thus, applying these findings to cancer therapy requires additional basic research to deepen our understanding of these mechanisms.

## Author contributions

KT-Drafting the manuscript, study concept, acquisition of data, supervision and coordination, and reading and approving the final manuscript.

## Conflicts of interest statement

The author declare that there are no conflicts of interest.
